# Supplementary Feeding Regulates Muscle Development of Oula Sheep (Tibetan Sheep, *Ovis aries*) Through Glucose Metabolism Pathway

**DOI:** 10.3390/ani15172626

**Published:** 2025-09-08

**Authors:** Yumeng Li, Yanhao Wang, Mingyi Yan, Sen Wu, Meng Liu, Sayed Haidar Abbas Raza

**Affiliations:** 1 Academy of Animal Science and Veterinary Medicine, Qinghai University, Xining 810016, China; liyum0520@163.com (Y.L.); wyh20000403a@163.com (Y.W.); ac17278321752@163.com (M.L.); 2 Plateau Livestock Genetic Resources Protection and Innovative Utilization Key Laboratory of Qinghai Province, Xining 810016, China; 3 Key Laboratory of Livestock and Poultry Genetics and Breeding on the Qinghai-Tibet Plateau (Qinghai), Ministry of Agriculture and Rural Affairs, Xining 810016, China; 4 State Key Laboratory of Biocontrol, School of Life Sciences, Sun Yat-sen University, Guangzhou 510275, China; haidar@mail.sysu.edu.cn

**Keywords:** Oula sheep, Tibetan sheep, supplementary feeding, longissimus dorsi muscle, RNA-seq, muscle development, carbohydrate metabolism

## Abstract

To investigate the genetic regulatory mechanism of supplementary feeding on muscle development in Oula sheep, we employed transcriptomic analysis to explore the differentially expressed genes (DEGs) in the longissimus dorsi muscle of Oula sheep at different ages under conditions of supplementary feeding and non-supplementary feeding, as well as the significantly enriched Gene Ontology (GO) terms and Kyoto Encyclopedia of Genes and Genomes (KEGG) signaling pathways of DEGs. Moreover, by combining with the method of weighted gene co-expression network analysis, we screened for the potential hub genes that might play crucial roles. The results demonstrated that the *CD4* and *ICAM1* genes and the PI3K-Akt signaling pathway might exert important functions during the lamb stage. At the growth stage, the *AGL*, *PGM2L1*, *PRKAA2*, *NEDD4*, and *GBE1* genes might serve as core genes to regulate the growth of skeletal muscle in Oula sheep after supplementary feeding through signaling pathways such as starch and sucrose metabolism and insulin signaling pathway. This outcome provides a molecular-level interpretation of the regulatory mechanism of supplementary feeding on muscle growth and development in Oula sheep at different ages, offering a theoretical basis for the further improvement of the meat quality of Oula sheep and the enhancement of the quality of livestock products in the Qinghai–Tibet Plateau region.

## Simple Summary

To systematically investigate the core factors regulating muscle development in the locally bred high-quality meat sheep Oula under the grassland grazing production model on the Qinghai–Tibet Plateau, this study included muscle tissue from Oula sheep under 2 nutritional estimation models: grazing production, and post-grazing supplementary feeding production, involving 6-month-old female lambs and 18-month-old female sheep. Transcriptome sequencing was conducted. The results revealed that the *CD4* and *ICAM1* genes and the PI3K-Akt signaling pathway may play important roles during the lamb stage. During the growth stage, the *AGL*, *PGM2L1*, *PRKAA2*, *NEDD4*, and *GBE1* genes may serve as core genes regulating skeletal muscle growth in Oula sheep after supplementary feeding through signaling pathways such as starch and sucrose metabolism and the insulin signaling pathway.

## 1. Introduction

The Oula sheep is a high-quality local breed found in high-altitude regions such as Qinghai, southern Gansu, and Tibet. This breed is characterized by its cold resistance and ability to thrive on coarse forage, with grazing being the primary feeding method. However, due to the unique geographical conditions of the Qinghai–Tibet Plateau, the alpine meadows provide insufficient forage resources during the long winter months to meet the growth needs of Oula sheep. Therefore, supplementary feeding in winter is crucial for the growth of Oula sheep. As a meat-type sheep, Oula sheep exhibit rapid growth and excellent fattening performance [1], and the meat is rich in protein and minerals, making it a high-quality meat product [2]. The proliferation and differentiation of muscle cells are regulated by various genes and transcription factors [3,4]. In mammals, skeletal muscle accounts for 40% to 60% of total body weight; thus, understanding the mechanisms underlying muscle cell growth and development can help us better regulate the growth rate of livestock muscle, enhancing the yield and quality of animal products. Studies indicate that the number of muscle fibers in mammals primarily increases during the fetal stage, with a constant number of muscle fibers postnatally. After birth, muscle growth is mainly due to an increase in fiber size and the differentiation of muscle fiber types [5]. Consequently, investigating the effects of supplementary feeding versus no supplementary feeding on the postnatal skeletal muscle development of Oula sheep is of critical importance.

With the development of second-generation sequencing technology, RNA-seq has been widely applied in the regulatory research of growth and development in livestock and poultry. A growing body of research has demonstrated that genes such as Myosin Light Chain (*MYL*) [6,7], B-cell translocation gene 2 (*BTG2*) [8], Fibroblast Growth Factor 6 (*FGF6*) [9], and Myostatin (*MSTN*) [7] play significant roles in muscle development in livestock and poultry. Wang et al. identified key regulatory genes in cattle muscle that are involved in signaling pathways such as cAMP, cGMP-PKG, and MAPK, which regulate growth and development [10]. Zhao et al. conducted transcriptomic sequencing of the longissimus dorsi muscle in goats and found that differentially expressed genes at various ages primarily function through pathways related to cellular energy [5]. Additionally, calcium ion binding, ECM-receptor interaction, and focal adhesion have been found to play critical roles in the mechanisms of fetal muscle differentiation in goats [11]. However, the mechanisms by which supplementary feeding influences muscle fiber development in meat sheep at different growth stages remain unclear.

Therefore, this study focuses on the Oula sheep as the research subject to investigate the genetic differences and regulatory mechanisms of muscle growth under supplementary feeding and non-supplementary feeding conditions. The findings aim to provide a theoretical basis for the genetic regulation and improvement of muscle growth and development in Oula sheep, as well as to offer new insights for the development of animal husbandry in the Qinghai–Tibet region.

## 2. Materials and Methods

### 2.1. Moral Statement

This trial was conducted with the approval of Qinghai Academy of Animal Husbandry and Veterinary Science (2025-QHMKY-008).

### 2.2. Sample Collection

A total of 30 healthy, weight-matched 6-month-old ewe lambs (with an average weight of 30.44 kg) and 30 healthy, weight-matched 18-month-old female Oula sheep (with an average weight of 41.06 kg) were selected from the same flock in the Henan Mongolian Autonomous County of Qinghai Province. The sheep were randomly assigned to two groups: a grazing group (un-supply) and a grazing plus supplementation group, with each group consisting of 5 replicates and 3 sheep per replicate. The experimental sheep were released for grazing at 09:00 and returned at 17:00 daily. The supplemented group received supplemental feeding upon return, with the dietary composition and nutritional ingredients shown in Table 1. The experimental duration was set to 60 days. At the end of the experiment, 5 sheep were randomly selected from each age group of both groups for slaughter, and samples of the longissimus dorsi muscle were collected.

### 2.3. RNA Extraction and Sequencing

RNA was extracted from the samples using the TRIzol reagent kit (TaKaRa) following the method described by Bao et al. [12]. The integrity of the extracted RNA was assessed, using Agilent 5400 detection of RNA purity and integrity, RNA purity and integrity meet the requirements for subsequent sequencing and research. cDNA libraries were subsequently constructed through reverse transcription. Quality control was performed on the cDNA libraries, and those meeting quality standards were sequenced using the Illumina platform. The raw sequencing data underwent quality control using FastQC, with low-quality reads being removed to obtain clean reads. The resulting clean reads were then aligned to the sheep reference genome using HISAT2 (2.2.1) software.

The data that support the findings of this study have been deposited into CNGB Sequence Archive (CNSA) of China National GeneBank DataBase (CNGBdb) with accession number CNP0004540.

### 2.4. Analysis of Differentially Expressed Genes

Differentially expressed genes were selected based on the criteria of Fold Change > 2 and *p*-value < 0.05. The identified differentially expressed genes underwent Gene Ontology (GO) and Kyoto Encyclopedia of Genes and Genomes (KEGG) enrichment analyses to reveal the primary biological functions and signaling pathways in which these genes are enriched. All the significant GO functions and KEGG pathways that are enriched were plotted, and the top 30 most significant ones were selected if there are more than 30.

### 2.5. Weighted Gene Co-Expression Network Analysis

A co-expression network was constructed for all differentially expressed genes using Weighted Gene Co-expression Network Analysis (WGCNA) by R package (1.71), allowing for the assessment of variability in gene expression among samples. The Pearson correlation coefficient was utilized to measure the correlation between genes, and these correlation values were subsequently weighted through power transformation to select an appropriate soft threshold. Based on the correlation heatmap between modules (the classified gene set), specific modules of interest were identified for further analysis. The genes from these selected modules were then intersected with the differentially expressed genes identified during the lamb and growth to find genes that were both significantly differentially expressed and present in the selected modules. Interaction analysis of these genes was performed using the STRING database (https://cn.string-db.org/, accessed on 27 December 2024) to construct a co-expression network, and the results were visualized using Cytoscape 3.10.3 software.

### 2.6. Validation by qPCR

Quantitative PCR (qPCR) was employed to validate the reliability of the transcriptome data. Primers were designed using the NCBI website, with ACTB serving as the reference gene for gene expression validation (Table 2).

## 3. Results and Analysis

### 3.1. Differential Gene Expression Profiles

**Figure 1:**
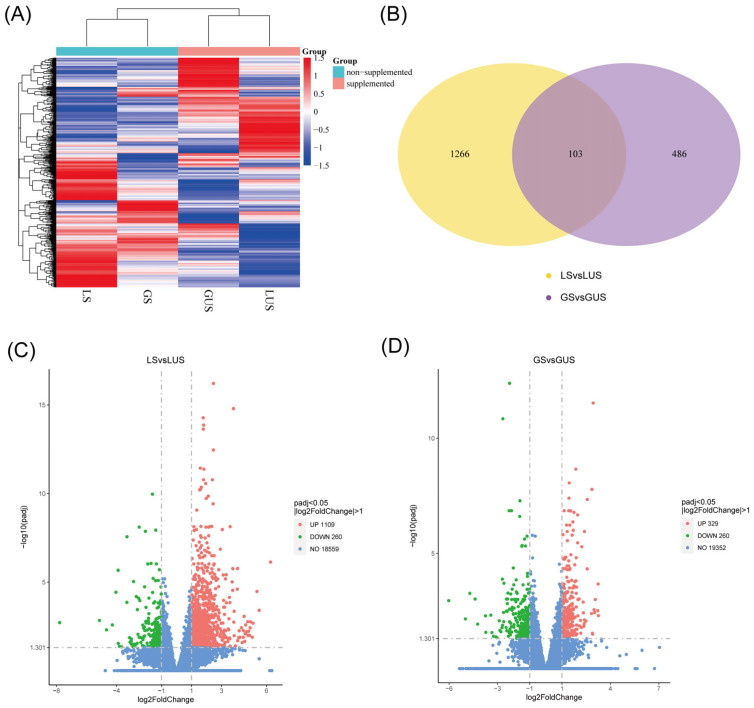
Analysis of differentially expressed genes with gene FPKM value. (**A**) gene expression cluster heat map. (**B**) differential expression gene Venn map. Yellow: the DEGs of LS Group vs. LUS Group; Light purple: the DEGs of GS Group vs. GUS Group. (**C**) LS vs. LUS DEGs volcano map. Green: the Downregulated DEGs; Red: the Upregulated DEGs. (**D**) GS vs. GUS DEGs volcano map. Green: the Downregulated DEGs; Red: the Upregulated DEGs.

The RNA quality control results are shown in Table 3 and gene expression group heatmap is shown in Figure 1A. The supplemented lambs and growing sheep clustered distinctly into two groups compared to their non-supplemented counterparts, indicating that supplementation does indeed impact gene expression. To analyze the genetic differences in muscle growth associated with supplementation in Oula sheep, we identified DEGs between the supplemented and non-supplemented groups at different stages. A total of 138 unique DEGs were identified in the LS (lamb supply) vs. LUS (lamb un-supply) comparison, and 122 unique DEGs were found in the GS (growth supply) vs. GUS (growth un-supply) comparison (Figure 1B). Overall, there were 1369 DEGs in the LS vs. LUS comparison, with 1109 upregulated and 260 downregulated genes. In the GS vs. GUS comparison, there were 589 DEGs, comprising 329 upregulated genes and 260 downregulated genes (Figure 1C,D).

### 3.2. GO and KEGG Enrichment Analysis of DEGs

**Figure 2:**
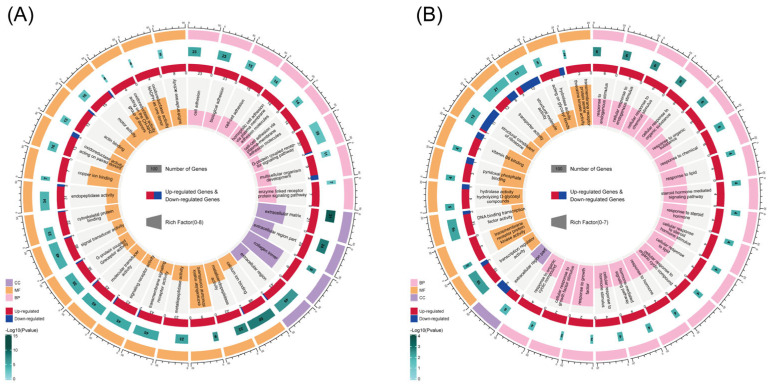
GO enrichment circle of differentially expressed genes. (**A**) LS vs. LUS DEGs GO enrichment. (**B**) GS vs. GUS DEGs GO enrichment. The 3 major functional categories of GO annotations—CC (Cellular Component), BP (Biological Process), and MF (Molecular Function)—are represented by purple, yellow, and pink in the outermost and innermost rings, respectively. The second layer from the right inward displays a heatmap with numerical labels indicating the significance level of each function (−Log10 (*p*-value)). The third layer displays Upregulated-DEGs and Downregulated-DEGs within that function, along with their respective counts, using red and blue colors and corresponding numerical values. The innermost bar chart indicates the total number of DEGs annotated with that GO functional category.

The DEGs in the LS vs. LUS comparison underwent GO functional annotation, resulting in a total of 717 GO terms, with 75 of these being significantly enriched. The significant GO terms were primarily concentrated in categories related to the extracellular matrix and signaling pathways. Figure 2A displays the top 30 most significant GO terms, which include extracellular matrix, extracellular matrix structural constituent, collagen trimer, copper ion binding, oxidoreductase activity (acting on the CH-NH2 group of donors), and oxidoreductase activity (acting on paired donors with the incorporation or reduction in molecular oxygen, where NAD(P)H serves as one donor and incorporates one atom of oxygen). These terms may play critical roles in regulating muscle development following supplementation during the lamb stage.

In the GS vs. GUS comparison, the DEGs were also subjected to GO functional annotation, revealing a high enrichment of terms such as transmembrane receptor protein kinase activity, response to growth factor, cellular response to growth factor stimulus, and transmembrane receptor protein serine/threonine kinase activity. These findings suggest that these terms may play significant regulatory roles in muscle growth and development during the growing stage of Oula sheep. Additionally, the DEGs were significantly enriched in functions related to carbohydrate and lipid metabolism, including hydrolase activity (hydrolyzing O-glycosyl compounds), response to lipid, cellular response to lipid, and hydrolase activity (acting on glycosyl bonds) (Figure 2B).

**Figure 3:**
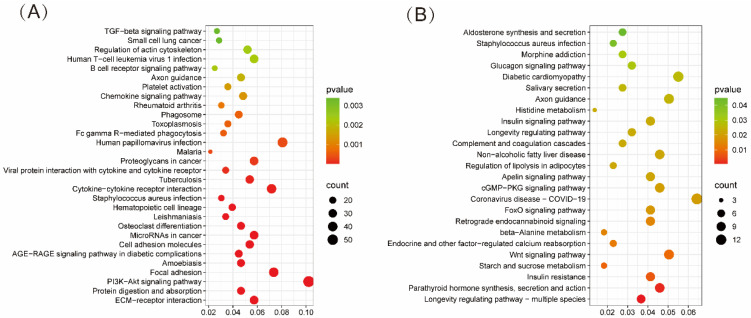
KEGG enrichment bubble plot. (**A**) KEGG enrichment of DEGs in the LS vs. LUS. (**B**) KEGG enrichment of DEGs in the GS vs. GUS. “count” represents the number of DEGs, the X-axis represents “GeneRatio”, and the Y-axis is sorted according to “*p*-value”.

KEGG enrichment analysis aids in further elucidating the potential functions of DEGs and the signaling pathways they participate in. The results indicate that DEGs in the LS vs. LUS comparison are significantly enriched in the PI3K-Akt signaling pathway (*p* = 1.73 × 10^−8^), which encompasses 57 DEGs closely related to the signaling and metabolic regulation of muscle cells. Notably, AKT plays a critical role in glucose metabolism, and its downstream effector molecule, mTOR, promotes cell proliferation and metabolism, being associated with protein synthesis. Additionally, pathways such as Protein digestion and absorption and ECM-receptor interaction were also significantly enriched (Figure 3A).

In the GS vs. GUS comparison, DEGs were primarily enriched in pathways related to starch and sucrose metabolism, beta-Alanine metabolism, regulation of lipolysis in adipocytes, insulin signaling pathway, and Histidine metabolism. These pathways are predominantly associated with the metabolism of nutrients and hormonal regulation (Figure 3B).

### 3.3. WGCNA Co-Expression Module

**Figure 4:**
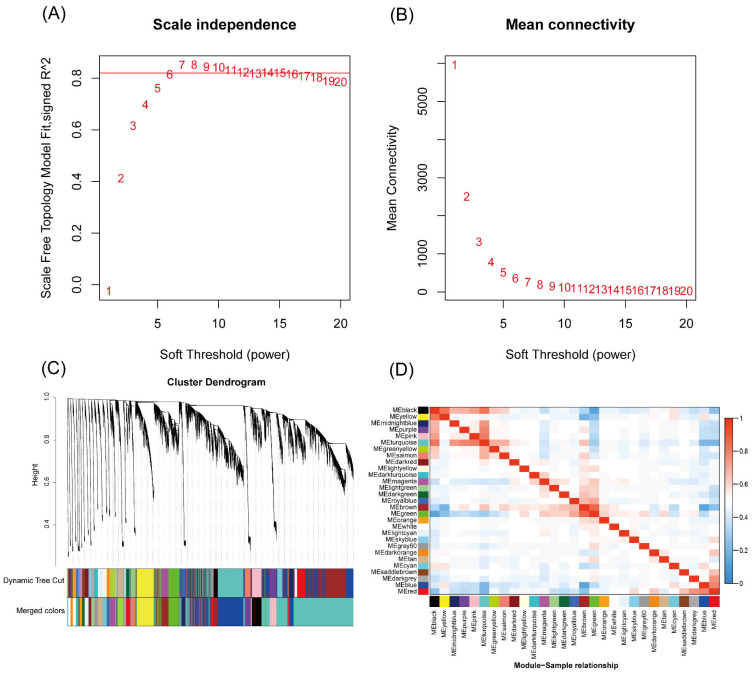
Scale-free fit index and average connectivity from WGCNA. (**A**) Soft threshold and cor-relation coefficient, where the x-axis represents the soft threshold (power value, β) and the y-axis shows the correlation between connectivity (k) and the probability (p(k)). The red line indicates the soft threshold and corresponding correlation coefficient for this study. (**B**) Soft threshold versus average connectivity of the network. (**C**) Hierarchical clustering dendrogram of modules. (**D**) Heatmap of correlations between modules.

Based on the scale-free fit index results, a soft threshold of β = 8 was selected, corresponding to the first value with R^2^ ≥ 0.8 (Figure 4A,B). Hierarchical clustering of the DEGs was performed using the dissimilarity between genes. Different modules were identified through dynamic cutting, and modules with a correlation coefficient greater than 0.75 were merged, resulting in a total of 30 modules (Figure 4C). Correlation analysis among the modules revealed that MEbrown and MEgreen exhibited relatively high correlations with other modules (Figure 4D).

### 3.4. Construction of Gene Co-Expression Network and Screening of Hub Genes

**Figure 5:**
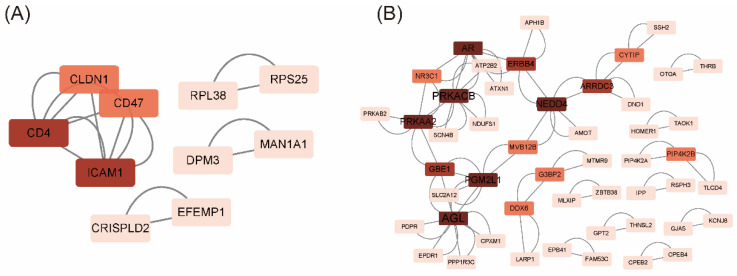
Common gene PPI network. The darker the color, the higher the core degree of the gene in the PPI network. (**A**) Intersection of significantly differentially expressed genes from the LS vs. LUS comparison with genes from the brown and green modules. CD4 and ICAM1 may be the hub gene in LS vs. LUS. (**B**) Intersection of significantly differentially expressed genes from the GS vs. GUS comparison with genes from the brown and green modules. AGL (Amylo-alpha-1, 6-glucosidase, 4-alpha-glucanotransferase), PRKACB (Protein kinase cAMP-activated catalytic subunit beta; Belongs to the protein kinase superfamily), PRKAA2 (Non-specific serine/threonine protein kinase), AR (Androgen receptor), NEDD4 (E3 ubiquitin-protein ligase), PGM2L1 (Phosphoglucomutase 2 like 1) may play a central regulatory role in the GS vs. GUS process.

To identify hub genes during the lamb and growing periods, we intersected the genes from the MEbrown and MEgreen modules with the DEGs from the LS vs. LUS and GS vs. GUS comparisons. This process allowed us to filter out genes that were significantly differentially expressed in both LS vs. LUS and GS vs. GUS and were present in the MEbrown and MEgreen modules. The resulting gene interaction network is presented in Figure 5.

During the lamb stage, *CD4* and *ICAM1* emerged as hub genes (Figure 5A). *CD4* primarily functions within the immune system, and *ICAM1* is associated with cell adhesion and signaling. In the growing stage, the top-ranking genes included *AGL*, *PGM2L1*, *PRKAA2*, *PRKACB*, *AR*, and *NEDD4*, along with *ERBB4* and *GBE1* (Figure 5B). The enzyme encoded by the *AGL* gene is involved in glycogen metabolism, the protein encoded by *PGM2L1* is related to glucose metabolism. *PRKAA2* regulates energy balance, and *PRKACB* plays a role in cellular signaling. *AR* is associated with hormonal signaling, and *NEDD4* participates in the ubiquitination of proteins, whereas *GBE1* is involved in starch and sucrose metabolism. Most of these genes are linked to nutrient metabolism and may regulate muscle growth and development through the metabolism and absorption of nutrients.

### 3.5. qPCR Validation

**Figure 6:**
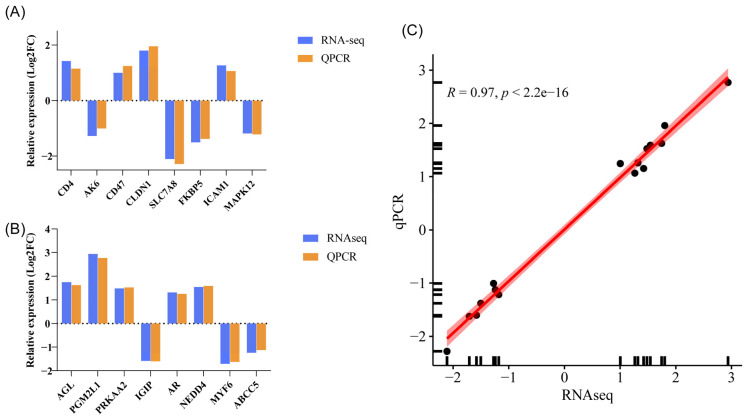
Verification of RNA-seq gene expression. (**A**) LS vs. LUS DEGs. (**B**) GS vs. GUS DEGs. (**C**) Correlation between RNA-seq and qPCR. A black dot represents a gene, and the red line indicates the linear fitting relationship between RNAseq and qPCR.

Eight DEGs from both the LS vs. LUS and GS vs. GUS comparisons were selected for qPCR validation to assess their relative expression levels. The results were consistent with the transcriptome data (Figure 6).

## 4. Discussion

Muscle growth is a critical factor influencing animal production performance. After birth, the development of muscle primarily involves increases in the diameter and length of muscle fibers, while the number of muscle fibers remains constant. *CD4*, *ICAM1*, *CD47*, and *CLDN1* are significantly differentially expressed genes identified in the LS vs. LUS comparison, all of which are related to signaling transduction. *CD4* is a glycoprotein that plays a role in recognizing antigens presented to T cell receptors and serves as a co-receptor expressed by T cells [13]. *ICAM1* is an adhesion molecule involved in inflammatory responses and the migration of immune cells; silencing this gene has been shown to alter the expression levels and concentrations of transcripts and metabolites associated with glycolysis [14]. Additionally, GO functions such as extracellular matrix and oxidoreductase activity, along with KEGG signaling pathways including ECM-receptor interaction, Protein digestion and absorption, and the PI3K-Akt signaling pathway, were significantly enriched. The PI3K-Akt signaling pathway is one of the crucial intracellular signaling pathways that enhances glucose utilization [15] and promotes glycogenolysis in the liver [16]. Furthermore, it can stimulate downstream mTOR effector molecules to facilitate cell proliferation and metabolism [17] while also playing a regulatory role in immune responses [18]. These findings suggest that supplementation during the lamb period can enhance the body’s immune response and the absorption utilization of nutrients such as glycogen, thereby promoting the growth and development of skeletal muscle. In this context, the *CD4* and *ICAM1* genes, along with the PI3K-Akt signaling pathway, may play key roles.

Previous research has shown that skeletal muscle fiber types can be categorized into slow-twitch oxidative (Type I), fast-twitch oxidative (Type IIa), and fast-twitch glycolytic (Type IIb) fibers. Muscles with a higher proportion of Type IIb fibers exhibit faster growth rates than those dominated by the other two fiber types [19]. In this study, we identified the genes *AGL*, *PGM2L1*, and *PRKAA2* during the growth phase of Oula sheep, all of which are directly involved in or indirectly regulate processes related to carbohydrate metabolism. AGL (Amylo-alpha-1,6-glucosidase, 4-alpha-glucanotransferase) is a multifunctional enzyme that plays a crucial role in glycolysis, encoding glycogen debranching enzyme (GDE) [20,21]. Complete or partial deficiency or mutation of AGL leads to autosomal recessive glycogen metabolism disorders, known as Type III glycogen storage disease [22,23]. PGM2L1 (Phosphoglucomutase 2 like 1) catalyzes various phosphotransferase reactions, including those involving (deoxy)ribose-1-phosphate and glucose-1-phosphate, contributing to the formation of intermediates such as 1,6-bisphosphate glucose [24], 1,6-bisphosphate mannose, and ribose-1,5-bisphosphate [25]. Notably, 1,6-bisphosphate glucose is directly involved in glycolysis, while ribose-1,5-bisphosphate can regulate the activity of phosphofructokinase, and 1,6-bisphosphate mannose may serve as a metabolic regulator [26]. These compounds are significantly linked to metabolic regulation, particularly carbohydrate metabolism. PRKAA2 (protein kinase AMP-activated alpha 2 subunit) encodes a catalytic subunit of AMPK, which regulates glycolysis by enhancing glucose uptake and promoting glycolytic pathways to generate more ATP [27]. Yang et al. also reported that AMPK activation can upregulate glycolysis [28]. Furthermore, studies have indicated that PRKAA2 may play a regulatory role in the immune response, metabolism, and nutrient absorption in sheep, potentially influencing their growth performance [29]. This research highlights the critical role of these genes in the metabolic processes that underpin muscle growth and development in sheep, providing valuable insights for improving livestock production efficiency.

Additionally, the DEGs identified in the comparison between GS and GUS were significantly enriched in several metabolic pathways related to nutrient utilization, including starch and sucrose metabolism, Beta-Alanine Metabolism, Histidine Metabolism, Regulation of Lipolysis in Adipocytes, and the insulin signaling pathway. In the starch and sucrose metabolism pathway, four annotated DEGs were identified: *AGL*, *PGM2L1*, *GBE1*, and *GAA*. The insulin signaling pathway showed significant enrichment with nine differentially expressed genes, among which are the hub genes *PRKAA2* and *PRKACB*. The starch and sucrose metabolism pathway involves the initial breakdown of disaccharides and polysaccharides into smaller molecules such as maltose, fructose, and glucose during digestion. Subsequently, glucose is further broken down through glycolysis to provide energy. When the glucose levels in the body rise, insulin secretion is upregulated as a regulatory response. Insulin is a key factor in glucose metabolism and lipid homeostasis, playing a critical role in glucose uptake and peripheral glucose regulation [30]. Furthermore, research has indicated a connection between insulin and longevity regulation [31]. The Longevity Regulating Pathway—Multiple Species was the most significantly enriched pathway identified in the KEGG analysis. This interconnectedness of the enriched KEGG signaling pathways aligns with the functional roles of the identified hub genes, most of which are associated with carbohydrate metabolism. These findings suggest that core genes such as *AGL*, *PGM2L1*, *PRKAA2*, *NEDD4*, and *GBE1* may function through pathways like starch and sucrose metabolism and the insulin signaling pathway to regulate skeletal muscle growth and development in Oula sheep following supplementation. These insights enhance our understanding of the molecular mechanisms underlying nutrient metabolism and muscle development, providing potential targets for improving livestock growth performance.

In summary, this study investigated the regulatory mechanisms underlying the effects of supplemental feeding on skeletal muscle growth and development in Oula sheep at different ages through mRNA expression profiling. The findings provide new insights into alleviating the slow growth of Tibetan sheep during winter in high-altitude regions, thereby contributing to the development of feed-efficient Tibetan sheep breeds. However, our research remains limited to the transcriptomic level. Further validation of the identified genes is necessary to screen for core genes regulating skeletal muscle development. Moreover, focusing solely on the genetic level is insufficient; studies at the protein expression level would serve as an important complement. There is a need to integrate genomic and proteomic analyses to comprehensively explore the impact of glycolytic differences on muscle development in Tibetan sheep.

## 5. Conclusions

This study investigated the changes in multiple genes related to muscle growth in Oula sheep under supplemented and non-supplemented conditions at different ages, as well as the potential regulatory mechanisms involved. It was found that during the lamb stage, *CD4* and *ICAM1* may enhance the immune response and regulate carbohydrate metabolism through a series of interactions, with the PI3K-Akt signaling pathway potentially playing a critical role in this process. In the growth phase, the genes *AGL*, *PGM2L1*, *PRKAA2*, *NEDD4*, and *GBE1* may function as core genes that regulate muscle growth and development in Oula sheep through signaling pathways such as starch and sucrose metabolism and the insulin signaling pathway.

## Figures and Tables

**Table 1:** Dietary composition and nutrient levels (dry matter basis).

Ingredients	Contents %	Nutrient Levels ^(2)^	Contents %
Corn	39.92	CP	15.09
Soybean meal	12.99	ME (MJ/kg)	2.62
Wheat bran	7.73	Ca	0.79
Rapeseed cake	6.93	TP	0.4
Limestone	1.42	Lys	0.67
Premix ^(1)^	1.01	Met	0.26
Dried corn stalk	30.00	NDF	33.34
Total	100.00	ADF	19.79

^(1)^ The premix provided the following per kg of diets: Cu 18 mg, Fe 50 mg, Mn 15 mg, Zn 16 mg, I 0.36 mg, Se 0.56 mg, Co 0.08 mg, VA 2000 IU, VD 342 IU, VE 200 IU. ^(2)^ Nutrient levels were calculated values.

**Table 2:** Primer sequences used for qPCR.

Gene	Accession No.	Forward (5′ → 3′)	Reverse (5′ → 3′)	Tm/°C	Size/bp
*ACTB*	XM_060405599.1	GTCAGCCGGTCCCATGGTC	ACACGGAGTACTTGCGCTC	61.4	102
*CD4*	NM_001129902.1	AACACTGAACTGAGCCATCGAGT	ACAGGTATAAGTCCCCGAGTCA	61.1	111
*ICAM1*	NM_001009731.1	GCAGTATCTCCTGTGACCGA	AGTTTGAGTAGCACAACGGGT	59.4	141
*CD47*	XM_060417299.1	TGTCCAAGCCCAGCAGTAAC	TCCTACGACGGCATCACTCT	60.3	104
*CLDN1*	NM_001185016.1	AGTGACAACATCGTGACGGC	TCAGCAAGGAGTCGAAGACTTT	60.3	106
*SLC7A8*	XM_004010333.6	AAGGTGTGCTAGAGAATGCCG	GTGACCCCGAGCTCAGCATA	61	109
*AK6*	NM_001285802.2	GAATGGCCACTGCACAATCA	ATCCACCTCCTAGTCTAACAGC	58.9	106
*FKBP5*	XM_042237057.2	GAACGAGTTCGAGTCAGCCA	CTCGTTGTGCTCCTTAGCCT	118	118
*AGL*	XM_012176992.4	AATTACCCACTTCCTGGAGAAGC	TGAAATGATCCAGCTTGTTGAAGAT	59.8	131
*PGM2L1*	XM_042233221.2	TTAGCCTTCGCCAGAACATCG	TGGCCAGATGACACACAGAGAG	61.4	109
*PRKAA2*	NM_001112816.1	AGCACGATGTCCACTGGATG	TCCAGCTGCTTCATAGCTCG	60.0	144
*AR*	NM_001308584.1	CAGCTGCTCCACCGATCTTAAA	AGATGGTCGAACTGCCTCCTA	60.5	179
*NEDD4*	XM_027971804.2	CACTCAGGGTTTTGATGGCG	CTGAGAAGCATCAATGGCCAAG	59.7	136
*MYF6*	XM_060411574.1	GAACCGGGATGTGCCCTG	GTCCACGATGGAAGAGAGGC	60.4	184
*IGIP*	XM_060415335.1	AAGAAGCGCAGTGTGTCGG	CAAGCCGGCTGATGCACAA	61.3	118

**Table 3:** RNA-seq sequencing data statistics.

Sample	Raw Reads Number	Clean Reads Number	Clean Reads Rate (%)	Clean Q30 Bases Rate (%)	GC Content (%)	Mapping Ratio (%)	Unique Mapping Ratio (%)
LUS1	46,648,844	42,782,078	91.71	93.42	48.45	93.81	80.77
LUS2	42,687,700	39,800,028	93.24	92.08	50.71	94.30	83.45
LUS3	48,102,646	47,210,960	98.15	93.07	52.03	95.93	84.65
LUS4	49,855,830	48,932,740	98.15	93.05	52.03	95.93	84.65
LUS5	49,225,048	47,874,618	97.26	93.50	52.05	95.87	84.82
LS1	41,212,104	38,519,372	93.47	91.99	51.8	94.64	83.34
LS2	46,013,906	43,545,542	94.64	92.17	49.36	95.43	81.43
LS3	45,748,468	43,155,138	94.33	92.39	52.42	94.87	84.31
LS4	45,241,120	41,792,798	92.38	92.5	49.01	94.89	80.92
LS5	46,274,008	43,830,938	94.72	92.06	51.92	94.95	83.81
GUS1	42,393,600	38,654,058	91.18	93.59	49.65	93.48	80.62
GUS2	49,635,300	45,807,122	92.29	93.37	49.22	95.06	78.09
GUS3	46,117,250	42,157,564	91.41	93.13	48.34	94.36	75.47
GUS4	43,868,834	43,798,780	99.84	92.93	49.18	93.68	77.80
GUS5	46,738,460	45,956,588	98.33	93.20	51.30	96.24	83.05
GS1	43,475,578	39,881,436	91.73	93.4	51.23	95.01	82.66
GS2	48,228,642	45,179,514	93.68	91.42	50.91	94.66	81.91
GS3	47,582,042	45,289,166	95.18	92.23	51.92	95.32	84.98
GS4	48,922,814	45,936,318	93.90	92.41	50.03	94.93	80.61
GS5	50,433,518	49,632,898	98.41	93.39	52.25	96.45	85.27

## Data Availability

All data generated or analyzed during this study are included in this published article.
